# HDL-C Levels Are Associated with Clinical Outcomes and Hospital Mortality in Critically Ill Patients with Sepsis: A Prospective Cohort Study

**DOI:** 10.3390/medicina62102002

**Published:** 2026-10-16

**Authors:** Hsin-Hsien Li, Tien-Ming Chan, How-Wen Ko, Chung-Shu Lee, Ping-Chih Hsu, Scott Chih-Hsi Kuo, Han-Chung Hu, Li-Chung Chiu

**Affiliations:** 1 Department of Respiratory Therapy, Chang Gung University College of Medicine, Taoyuan 33302, Taiwan; hsinhsien@mail.cgu.edu.tw; 2 Department of Thoracic Medicine, Chang Gung Memorial Hospital, Linkou Branch, Taoyuan 33305, Taiwan; q12040@cgmh.org.tw (H.-W.K.); jraacyk@adm.cgmh.org.tw (C.-S.L.); 8902049@cgmh.org.tw (P.-C.H.); r5245@cgmh.org.tw (S.C.-H.K.); h3226@cgmh.org.tw (H.-C.H.); 3 Division of Rheumatology, Allergy, and Immunology, Chang Gung Memorial Hospital, Linkou Branch, Taoyuan 33305, Taiwan; b9005036@cgmh.org.tw; 4 College of Medicine, Chang Gung University, Taoyuan 33302, Taiwan; 5 Department of Thoracic Medicine, New Taipei Municipal TuCheng Hospital and Chang Gung University, Taoyuan 33302, Taiwan

**Keywords:** critical illness, sepsis, acute respiratory distress syndrome, conventional lipid parameters, high-density lipoprotein cholesterol, outcomes, mortality

## Abstract

*Background and Objectives*: Metabolic perturbations frequently occur during the acute phase of critical illness and may disrupt lipid homeostasis. Acute respiratory distress syndrome (ARDS) is a severe condition and is associated with high mortality in critically ill patients with sepsis. This study aimed to evaluate the association between conventional plasma lipid parameters and clinical outcomes in critically ill sepsis patients. *Materials and Methods*: We conducted a prospective observational cohort study of critically ill sepsis patients in Taiwan between October 2020 and July 2025. Conventional plasma lipid parameters and clinical variables were measured at intensive care unit admission. Clinical outcomes were compared between patients stratified by plasma HDL-C levels. *Results*: A total of 285 critically ill patients with sepsis were included. Pneumonia was the presumed source of infection in 91% of patients, and 78% met the criteria for ARDS. The overall hospital mortality rate was 37.9%. Patients with ARDS had significantly higher C-reactive protein (CRP) and interleukin-6 (IL-6) levels, higher risk of organ failure (i.e., APACHE II and SOFA scores), and lower plasma HDL-C levels (all *p* < 0.05). Non-survivors exhibited significantly greater organ failure severity and lower HDL-C levels compared with survivors (all *p* < 0.05). Patients with low HDL-C (≤17.5 mg/dL; *n* = 106, 37.2%) had significantly higher CRP and IL-6, greater organ failure severity, and increased 28-, 60-, 90-day, and hospital mortality, compared with those with high HDL-C (> 17.5 mg/dL; *n* = 179, 62.8%) (all *p* < 0.05). In multivariable analyses, lower HDL-C were independently associated with hospital mortality. *Conclusions*: Lower HDL-C levels were associated with hospital mortality in critically ill patients with sepsis, most of whom had pneumonia and concomitant ARDS. These findings suggest that HDL-C may have prognostic value in this selected critically ill population, although external validation in broader sepsis populations is warranted. An easily accessible and useful for early risk stratification.

## 1. Introduction

Critical illness encompasses life-threatening conditions that require prompt intensive monitoring, support, and treatment of vital organ functions. Sepsis and acute respiratory distress syndrome (ARDS) represent two of the most common causes of critical illness among patients admitted to the intensive care unit (ICU) and are major contributors to multiple organ failure, with persistently high morbidity and mortality.

ARDS is a life-threatening form of acute hypoxemic respiratory failure characterized by oxidative stress, dysregulated airway and systemic inflammatory cascades and immune activation [1,2,3,4]. To date, no effective pharmacotherapies have been established, and lung-protective mechanical ventilation remains the cornerstone of evidence-based management for ARDS, with demonstrated benefits in improving clinical outcomes [5]. Despite significant advances in ARDS management, morbidity and mortality remain unacceptably high. Early identification of potential risk factors and prognostic determinants during the course of critical illness is therefore essential and may offer opportunities to reduce the burden of ARDS and improve patient outcomes.

Lipid synthesis, transport, and metabolism are frequently disrupted during the acute phase of critical illness, thereby affecting immune function, hormone and vitamin production, as well as cell membrane integrity and signaling pathway. High-density lipoprotein (HDL) is a complex lipoprotein particle that exerts antioxidant, anti-inflammatory, anti-apoptotic, antithrombotic, and immunomodulatory properties [6,7]. Hypocholesterolemia commonly occurs during critical illness and has been associated with disease severity and adverse clinical outcomes. However, the underlying pathophysiological mechanisms are multifactorial, complex, and not yet fully elucidated.

Systemic inflammation and oxidative stress, which are hallmarks of critical illness with sepsis and key contributors to ARDS pathogenesis, may induce structural and compositional modifications of HDL particles, resulting in dysfunctional HDL with impaired antioxidant and anti-inflammatory properties. Such alterations may reduce the protective functions of HDL and contribute to endothelial dysfunction, dysregulated immune responses, and organ injury. Although high-density lipoprotein cholesterol (HDL-C) is commonly used as a quantitative measure of HDL, it may also reflect broader alterations in HDL metabolism and functionality during critical illness.

The relationship between HDL-C levels and clinical outcomes in critically ill sepsis patients, particularly among those with and without ARDS, remains incompletely understood. Therefore, this study aimed to investigate the association between conventional lipid parameters, especially HDL-C, and clinical outcomes in critically ill patients with sepsis.

## 2. Materials and Methods

### 2.1. Study Design and Patients

This prospective observational study was conducted between October 2020 and July 2025 in the medical ICUs of a tertiary referral center, Chang Gung Memorial Hospital (CGMH), Linkou branch, Taiwan. The source population comprised patients admitted to the medical ICUs during the study period. The target population consisted of critically ill patients with sepsis associated with pneumonia (bacterial or viral pneumonia) or extrapulmonary sepsis who were eligible for study enrollment. Sepsis was defined according to the Third International Consensus Definitions for Sepsis and Septic Shock (Sepsis-3) as life-threatening organ dysfunction caused by a dysregulated host response to infection [8]. Shock was defined as a clinical condition characterized by inadequate tissue perfusion and oxygen delivery, regardless of etiology. Acute kidney injury (AKI) was defined according to the KDIGO criteria based on serum creatinine levels and/or urine output [9]. The inclusion criteria of ARDS was defined according to the Berlin criteria [10]. Patients who met the Berlin criteria at ICU admission were classified into the ARDS group, whereas those who did not meet the criteria were classified into the non-ARDS group. Participants were further categorized according to survival status (survivors vs. non-survivors) and HDL-C levels (high vs. low HDL-C groups based on the optimal ROC-derived cutoff value).

Eligible patients were prospectively enrolled after informed consent had been obtained from the patients or their legally authorized representative. The exclusion criteria were: (1) age < 20 years; (2) a moribund condition with refractory multiple organ failure and an expected survival of less than 24 h; and (3) inability to obtain informed consent. Due to operational and logistical limitations, not all subjects at ICU admissions were screened for eligibility. Enrollment was dependent on research staffing and operational feasibility, resulting in only a subset of potentially eligible ICU patients underwent active screening and enrollment assessment during the study period. This study was conducted in accordance with the Declaration of Helsinki, and ethical approval was obtained from the Institutional Review Board of CGMH for Human Research (CGMH IRB No. 202000760A3, 202100595A3, 202201833A3, and 202300897A3).

### 2.2. Data Collection

Demographic characteristics and underlying comorbidities were recorded for all participants. Most patients had been diagnosed with sepsis before ICU admission and were admitted because of sepsis-related critical illness. Blood samples were collected within 12 h of ICU admission in 6 mL plastic tubes containing K_2_EDTA as an anticoagulant (BD, Franklin Lakes, NJ, USA), and analyses were performed in the institutional biochemistry laboratory. Serum concentrations of high-density lipoprotein cholesterol (HDL-C), low-density lipoprotein cholesterol (LDL-C), total cholesterol, and triglycerides were measured using automated enzymatic assays. HDL-C was measured using a direct enzymatic method, which selectively measures HDL cholesterol using a surfactant-based reaction system followed by enzymatic color development. LDL-C was directly measured using a selective solubilization method, which selectively solubilizes LDL cholesterol while suppressing reactions involving other lipoproteins. Total cholesterol was measured using an enzymatic oxidation method, in which cholesterol esters are hydrolyzed and the resulting free cholesterol undergoes an enzymatic reaction to generate a colored product. Triglycerides was measured using an enzymatic method with free-glycerol elimination, in which triglycerides are hydrolyzed enzymatically and the resulting reaction produces a colored product proportional to the triglycerides concentration. The absorbance of the reaction products was measured photometrically, and lipid concentrations were determined using the corresponding assay calibration procedures. Because the exact time of sepsis onset or recognition was not systematically recorded, the precise interval between sepsis onset and blood sampling could not be determined. Clinical and laboratory variables were obtained, including interleukin-6 (IL-6); conventional plasma lipid parameters, including HDL-C, LDL-C, total cholesterol, and triglycerides; recent statin use within the preceding six months; Acute Physiology and Chronic Health Evaluation II (APACHE II) score; and Sequential Organ Failure Assessment (SOFA) score. The dates of hospital and ICU admission, mechanical ventilator initiation and liberation, ICU and hospital discharge, and time of death were recorded. In addition, the presence of shock, use of vasopressor, and requirement for renal replacement therapy during the ICU stay were documented.

### 2.3. Outcome Measurements

Hospital mortality was defined as death from any cause during the index hospitalization. Mortality outcomes included 28-day, 60-day, and 90-day all-cause mortality, with the date of ICU admission defined as time zero for these outcomes. Thus, 28-, 60-, and 90-day mortality were defined as death occurring within 28, 60, and 90 days, respectively, after ICU admission. For patients discharged before the end of these follow-up periods, survival status was ascertained using available subsequent medical records and mortality records. Complete follow-up information for the 28-day, 60-day, and 90-day mortality outcomes was available for all included patients. Additional clinical outcomes included the incidence of shock, use of vasopressor, development of AKI and requirement for renal replacement therapy after ICU admission and HDL-C sampling, duration of mechanical ventilation, length of stay in the ICU, and total hospital length of stay.

### 2.4. Statistical Analysis

Comparisons between groups were performed using analysis of variance or the Student’s *t* test for normally distributed variables, and the Kruskal–Wallis test or Mann–Whitney U test for non-normally distributed variables, as appropriate. All continuous variables were reported as mean (standard deviation) for normally distributed variables or median (interquartile range) for non-normally distributed variables. Categorical variables were reported as frequency (proportion) and were compared using the chi-square test for equal proportions or Fisher’s exact test. Receiver operating characteristic (ROC) curve analysis was performed to evaluate the ability of HDL-C measured at ICU admission to discriminate between survivors and non-survivors. The optimal HDL-C cutoff value was determined using the Youden index (sensitivity + specificity − 1). The ROC-derived cutoff was subsequently used to dichotomize HDL-C levels in outcome analyses. Because the cutoff value was derived and evaluated in the same cohort, it was considered exploratory. Univariate analysis was examined to identify the risk factors associated with hospital mortality in the first step, followed by the construction of multivariate logistic regression models. To control for potential covariances, all candidate variables were selected based on clinically driven selection and included in the multivariable model using the Enter method. The results were expressed using odds ratio (OR) and 95% confidence interval (CI). HDL-C was analyzed primarily as a continuous variable in the multivariable logistic regression models. Sensitivity analyses were additionally performed by separately incorporating C-reactive protein (CRP), IL-6 and PaO_2_/FiO_2_ into the primary model to evaluate the robustness of the association between HDL-C and clinical outcomes. Missing data were handled using a complete-case approach for each regression model. Models incorporating IL-6 were restricted to patients with available IL-6 measurements and complete data for all other variables included in the model. Kaplan–Meier method was employed to estimate the cumulative incidence of hospital survival for each group and the difference in survival curve was assessed by log-rank test. All statistical analyses were performed using SPSS version 29.0 (IBM Inc., Armonk, NY, USA), and a two-sided *p* value less than 0.05 was considered statistically significant.

## 3. Results

**Figure 1:**
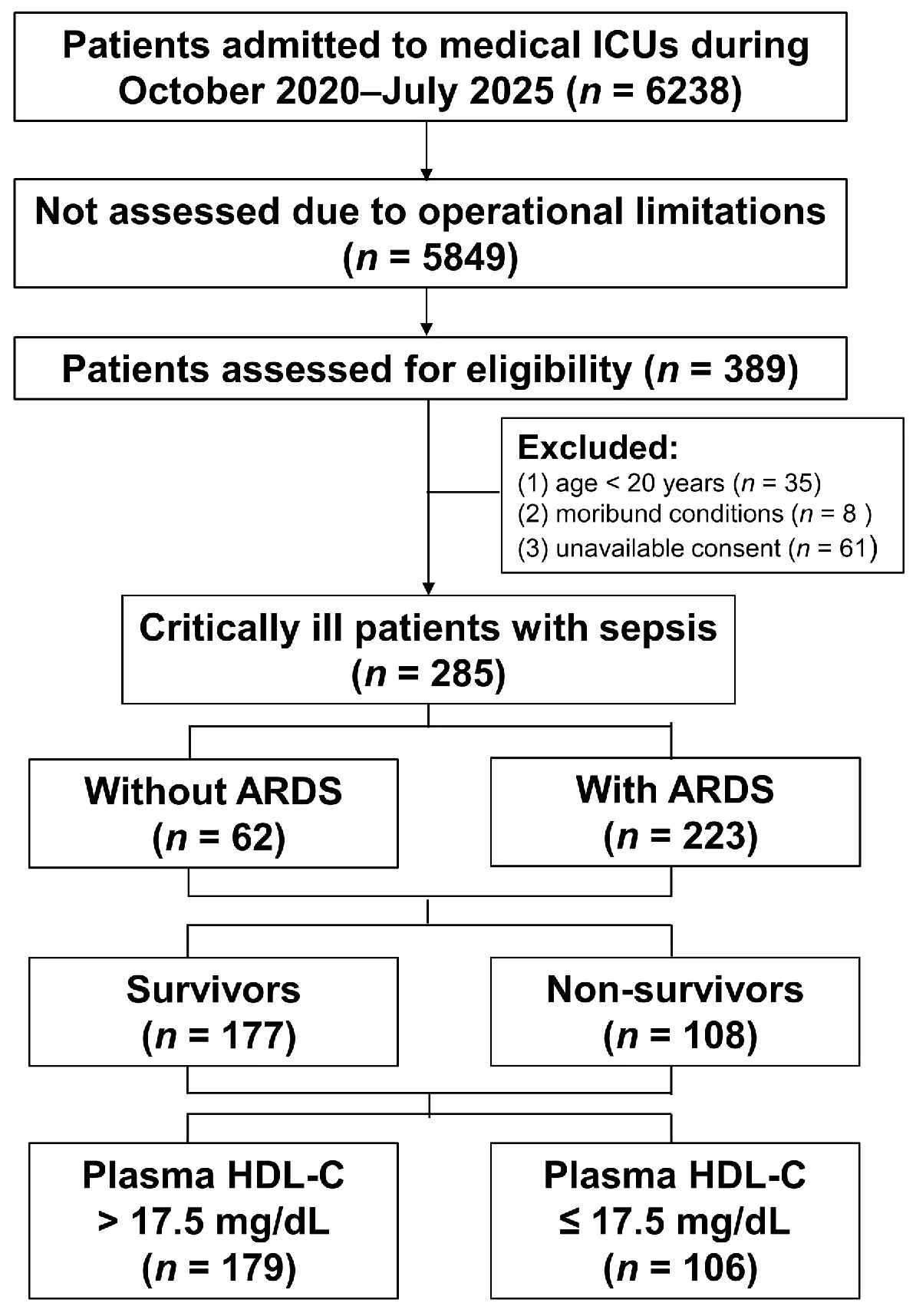
Flowchart of the enrollment of critical ill patients with sepsis. ARDS: acute respiratory distress syndrome; HDL-C: high-density lipoprotein cholesterol; ICU: intensive care unit.

During the study period, 6238 patients admitted to the medical ICUs were screened for potential study enrollment. Of these, 5849 were not assessed for study enrollment because active screening depended on research staffing and operational limitations. The remaining 389 patients underwent eligibility assessment, of whom 104 were excluded because of age < 20 years (*n* = 35), moribund condition (*n* = 8) or unavailable informed consent (*n* = 61). Finally, 285 critically ill patients with sepsis were enrolled in the study. Of these, 62 patients without ARDS and 223 patients with ARDS at ICU admission were included in the final analysis (Figure 1). The overall all-cause in-hospital mortality among the study population was 37.9%.

### 3.1. Comparisons Between Critically Ill Sepsis Patients with and Without ARDS

As shown in Table 1, there were no significant differences in age or gender distribution between patients with and without ARDS. Body mass index (BMI) was significantly higher in the ARDS group than in the non-ARDS group. The proportion of bacterial pneumonia was significantly higher in the ARDS group. Underlying comorbidities were generally comparable between the two groups.

Both APACHE II and SOFA scores were significantly higher in the ARDS group than in the non-ARDS group (both *p* < 0.05). Inflammatory markers including CRP and IL-6 were also significantly elevated in patients with ARDS (all *p* < 0.05).

Among conventional lipid parameters, levels of HDL-C, LDL-C, and total cholesterol were significantly lower in the ARDS group compared with the non-ARDS group (all *p* < 0.05), whereas triglyceride levels were significantly higher in the ARDS group (*p* = 0.008). Statin use was observed in 7.6% of patients with ARDS, whereas no patients without ARDS had received statin therapy. Patients in the ARDS group had significantly higher rates of shock and use of vasopressor. All-cause in-hospital mortality was significantly higher in the ARDS group than in the non-ARDS group (43.0% vs. 19.4%, *p* = 0.001).

### 3.2. Comparisons Between Critically Ill Survivors and Non-Survivors

As shown in Table 2, no significant differences in age or gender distribution were observed between survivors and non-survivors. BMI was significantly higher among survivors. The proportion of immunocompromised status was more prevalent among non-survivors (*p* < 0.05).

Both APACHE II and SOFA scores were significantly higher in non-survivors than in survivors (both *p* < 0.05). There were no significant differences in CRP or IL-6 levels between survivors and non-survivors. Non-survivors also exhibited more severe hypoxemia, as reflected by lower PaO_2_/FiO_2_ ratios (*p* = 0.010).

Regarding conventional lipid parameters, HDL-C and total cholesterol levels were significantly lower in non-survivors than in survivors (all *p* < 0.05). The values of lower LDL-C and higher triglyceride was detected in non-survivors; however, these differences did not reach statistical significance. Statin use did not differ significantly between the two groups. Non-survivors had significantly higher rates of shock and use of vasopressor. The proportions of mechanical ventilation use and ARDS occurrence were both significantly higher among non-survivors.

### 3.3. Comparisons Between Critically Ill Sepsis Patients with High and Low Plasma HDL-C Levels

The area under the ROC curve for HDL-C in predicting hospital mortality was 0.609 (95% CI: 0.542–0.676). The exploratory optimal cutoff value, determined by the maximum Youden index, was 17.5 mg/dL, with a sensitivity of 0.491 and a specificity of 0.701. The corresponding AUCs for the SOFA and APACHE II scores were 0.765 and 0.685, respectively. Participants were stratified into a high HDL-C group (*n* = 179, 62.8%) and a low HDL-C group (*n* = 106, 37.2%) based on the optimal cutoff value of 17.5 mg/dL at ICU admission (Table 3).

Patients in the high HDL-C group were older than the low HDL-C group. APACHE II and SOFA scores were significantly higher in the low HDL-C group than in the high HDL-C group (both *p* < 0.05). The low HDL-C group also exhibited significantly higher levels of inflammatory markers, including CRP and IL-6, and more severe hypoxemia, as reflected by lower PaO_2_/FiO_2_ ratios (all *p* < 0.05).

Regarding conventional lipid parameters, lower levels of HDL-C, LDL-C, and total cholesterol, along with higher triglyceride levels, were observed in the low HDL-C group compared with the high HDL-C group, with all differences reaching statistical significance (all *p* < 0.05).

### 3.4. Clinical Outcomes of Critically Ill Sepsis Patients Stratified by Plasma HDL-C Level

The 28-, 60-, and 90-day mortality, as well as all-cause in-hospital mortality, were all significantly higher in the low HDL-C group (all *p* < 0.05). Patients in the low HDL-C group also had significantly higher rates of shock, use of vasopressor, and AKI (all *p* < 0.05), whereas the requirement for renal replacement therapy did not differ significantly between the two groups. No significant differences were observed between the groups in the duration of mechanical ventilation, ICU length of stay, or total hospital length of stay (Table 4).

### 3.5. Factors Associated with Hospital Mortality

After adjusting for potential confounders, multivariable logistic regression analyses demonstrated that immunocompromised status, lower BMI, higher SOFA score, and lower plasma HDL-C levels were independently associated with in-hospital mortality. HDL-C was analyzed as a continuous variable and was independently associated with hospital mortality (adjusted OR 0.955 [95% CI 0.921–0.990], *p* = 0.013) (Table 5). The association remained statistically significant in sensitivity analyses with additional adjustment for CRP, IL-6, and PaO_2_/FiO_2_ (Table 6).

**Figure 2:**
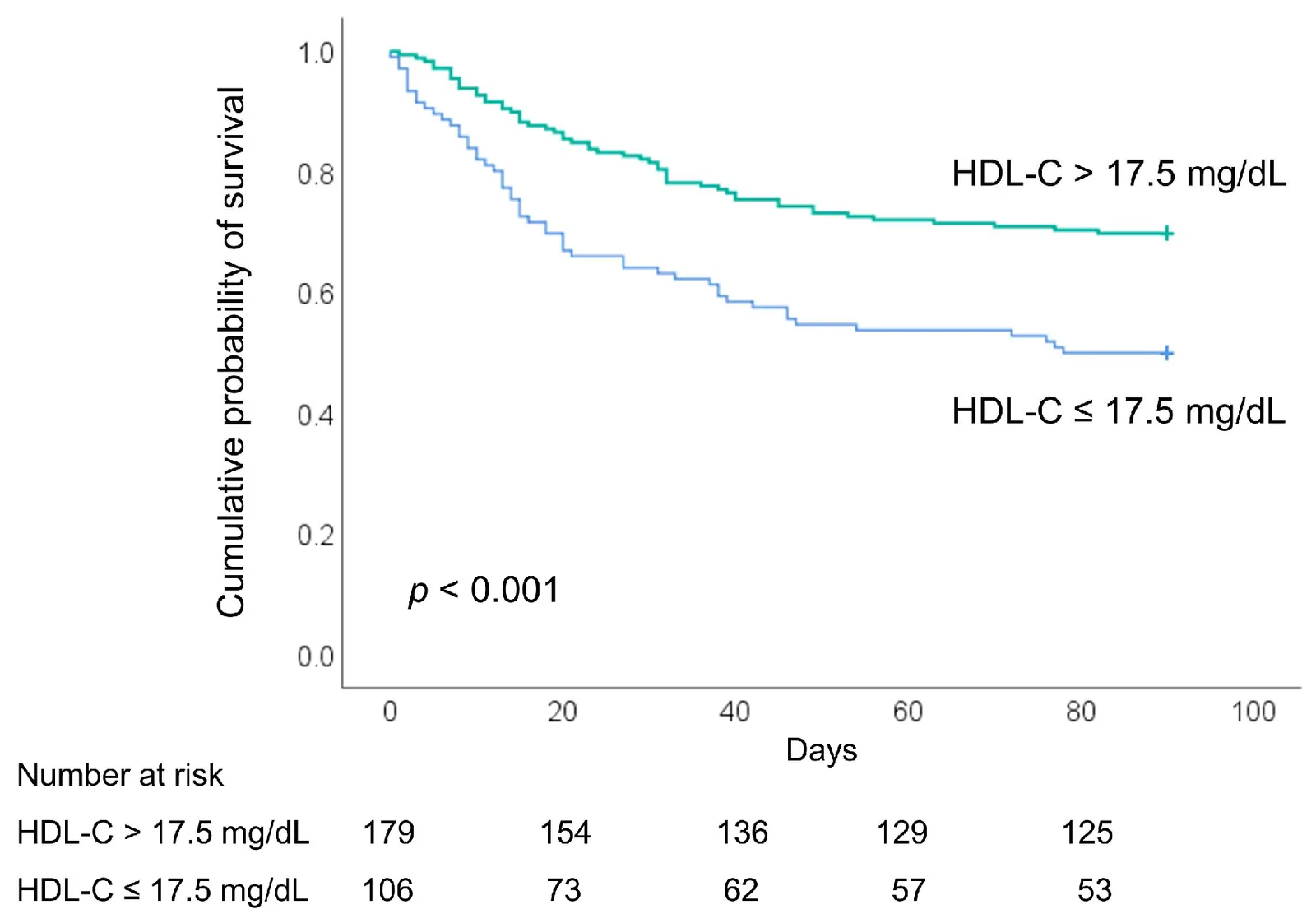
Kaplan–Meier 90-day survival curves for critically ill patients stratified by plasma HDL-C levels using a cutoff of 17.5 mg/dL measured at ICU admission. Time zero was defined as the date of ICU admission. Numbers at risk are shown below the survival curves. HDL-C: high-density lipoprotein cholesterol; ICU: intensive care unit.

Furthermore, the 90-day survival rate was significantly higher in the high HDL-C group (>17.5 mg/dL) compared with the low HDL-C group (≤17.5 mg/dL) (69.8% vs. 50%, *p* < 0.001, log-rank test) (Figure 2).

## 4. Discussion

The key finding of this prospective study is that plasma HDL-C was independently associated with in-hospital mortality in critically ill patients with sepsis, particularly in patients with pneumonia and concomitant ARDS. Our findings suggest that plasma HDL-C measured at ICU admission may serve as an easily accessible and useful for early risk stratification in this population that requires external validation.

Metabolic derangements in glucose, amino acid, and lipid metabolism occur during both acute and prolonged critical illness and are closely associated with disease severity and clinical outcomes [11,12,13,14]. Critical illnesses, particularly sepsis, are characterized by a hypermetabolic and hypercatabolic state that disrupts lipid homeostasis. This disruption is marked by an immediate and sustained decline in plasma HDL-C, LDL-C, and total cholesterol levels, the magnitude of which correlates with the intensity of inflammation, disease severity, and mortality, whereas hypertriglyceridemia appears to have limited prognostic value. Potential underlying mechanisms include reduced dietary intake and intestinal fat absorption, decreased lipid synthesis, impaired cholesterol transport, increased metabolic consumption, and enhanced toxin scavenging [15,16,17,18].

HDL exerts multiple biological functions, including promoting macrophage cholesterol efflux and reverse cholesterol transport from peripheral tissues to the liver, maintaining endothelial function by stimulating nitric oxide production, and providing antioxidative effects through the inhibition of LDL oxidation. In addition, HDL has anti-inflammatory, anti-apoptotic, antithrombotic, and immunomodulatory properties. Notably, HDL particles can bind and neutralize lipopolysaccharide and circulating cytokines via scavenger receptor class B type 1, the principal hepatic HDL receptor [6,7,17,19,20,21,22,23,24,25,26].

Plasma HDL-C levels have been shown to correlate with the severity of organ failure and clinical outcomes in patients with critical illness, including those with sepsis and septic shock [27,28,29,30,31,32]. Systematic reviews and meta-analyses reported an association between lower circulating HDL-C levels and mortality in patients with sepsis and critical illness [32,33]. Previous studies also revealed that hypolipidemia, including lower HDL-C levels, is associated with greater disease severity and mortality in patients with COVID-19 [34,35]. HDL-C levels may decrease during the early phase of sepsis and subsequently increase with clinical recovery, while persistently low HDL-C levels were associated with mortality [36,37,38]. However, whether conventional lipid parameters differ between critically ill sepsis patients with and without ARDS remains unclear. In the present study, patients who had ARDS at ICU admission exhibited significantly higher levels of inflammatory markers, including CRP and IL-6, as well as greater disease severity, reflected by higher APACHE II and SOFA scores, compared with those without ARDS. Notably, patients with ARDS also demonstrated significantly lower levels of HDL-C, LDL-C, and total cholesterol (all *p* < 0.05).

In multivariable regression analyses, HDL-C remain independently associated with in-hospital mortality beyond inflammatory (CRP and IL-6) and severity markers (PaO_2_/FiO_2_). HDL could provide additional biological properties including regulation of lipid metabolism and transport, hormone synthesis, endotoxin neutralization, modulation of cytokine release, and maintenance of vascular homeostasis. This complementary role may explain that HDL reflect pathophysiological processes beyond systemic inflammation and why HDL-C remained independently associated with adverse outcomes even after adjustment for inflammatory and severity markers. Our findings suggest that critically ill sepsis patients may experience profound dysregulation of inflammatory and immune responses, accompanied by progressive organ dysfunction during the acute phase of critical illness. These processes could lead to alterations in lipid metabolism, including increased lipoprotein consumption for endotoxin neutralization and enhanced utilization of cholesterol for steroidogenesis and other metabolic demands associated with the stress response to critical illness, thereby contributing to hypocholesterolemia and are at an increased risk of mortality. Although lower HDL-C levels were independently associated with hospital mortality after adjustment for disease severity, adding HDL-C to the SOFA score did not improve the model’s discriminatory performance. The AUC was 0.765 for the SOFA-only model and 0.764 for the combined model. These findings suggest that the independent association between HDL-C and mortality does not necessarily translate into incremental prognostic discrimination beyond an established severity score. Therefore, the clinical utility of HDL-C as an additional prognostic marker remains uncertain.

In addition, previous studies have reported that underweight status is associated with an increased risk of mortality, whereas overweight and obesity are associated with a lower risk of mortality among critically ill patients [39,40]. Consistent with these findings, our multivariable regression analyses demonstrated that a lower BMI was independently associated with increased hospital mortality. However, the mean BMI of our study population (23.6 kg/m^2^) was within the normal range, and patients were not further stratified according to BMI categories.

A previous study showed that an HDL-C level below the cutoff of 20 mg/dL was significantly associated with mortality in patients with severe sepsis. Similarly, another study revealed that an HDL-C level below a cutoff of 25.1 mg/dL was significantly associated with multiple organ dysfunction and death in patients with suspected sepsis [27,36]. In our study, critically ill sepsis patients with low plasma HDL-C levels (≤17.5 mg/dL) exhibited significantly higher inflammatory markers, including CRP and IL-6, more severe hypoxemia, greater organ failure severity, and lower levels of HDL-C, LDL-C, and total cholesterol compared with those with higher HDL-C levels. In addition, patients in the low HDL-C group experienced worse clinical outcomes, including a significantly higher risk of all-cause in-hospital mortality, shock, vasopressor use, and AKI (all *p* < 0.05). These findings suppose that critically ill sepsis patients with more exaggerated inflammatory responses and multiple organ dysfunction may consume greater amounts of HDL particles due to their endotoxin-scavenging and anti-inflammatory properties, leading to reduced circulating HDL-C levels. This process may, in turn, contribute to poorer clinical outcomes.

Although high HDL-C concentrations have traditionally been considered protective against cardiovascular and inflammatory diseases, their causal role remains uncertain, and extremely elevated levels may be detrimental to human health. Emerging evidence suggests a U-shaped relationship between HDL-C levels and clinical outcomes, with both low and excessively high HDL-C concentrations (>80 mg/dL in men and >100 mg/dL in women) associated with increased risk of adverse outcomes and all-cause mortality—a phenomenon referred to as the “HDL cholesterol paradox” [41,42,43]. The structure and function of HDL particles are highly heterogeneous and complex. Accordingly, the biological effects of HDL-C may depend not only on its circulating quantitative concentration but also on qualitative characteristics, including particle size and number, density, charge and shape, functional assay, apolipoprotein composition, and cholesterol efflux capacity [19,22,25,26,31,44,45,46]. These considerations highlight that HDL-C concentration alone may not fully capture its functional capacity, and further investigation into HDL functionality may provide deeper insights into its role in critical illness.

This study has several limitations. First, this was a single-center observational cohort study conducted at a tertiary medical center in Taiwan, which may limit the generalizability of our findings. As a tertiary referral medical center, our institution may care for patients with greater illness severity and a different case-mix than other populations and institutions. Moreover, the lack of internal and external validation may have reduced the robustness and external validity of the study findings. Second, as patients were enrolled during the acute phase of critical illness characterized by a hypercatabolic state, conventional plasma lipid parameters were measured only at ICU admission; thus, dynamic changes over time were not assessed. Therefore, we could not evaluate temporal changes in HDL-C during critical illness or determine whether serial measurements provide incremental prognostic value beyond a single baseline measurement. Third, the study has potential selection bias because only a subset of medical ICU admissions underwent formal eligibility assessment and enrollment, owing to operational and staffing limitations. Because clinical characteristics and outcomes were not systematically available for patients who were not assessed or enrolled, we could not evaluate differences between enrolled and non-enrolled patients. Therefore, the enrolled cohort may not be representative of the overall ICU population, which may limit the generalizability of our findings. Most enrolled patients had pneumonia (91%), and 78% met the criteria for ARDS. The proportion of ARDS cases was relatively high, which warrants cautious interpretation of the results. Accordingly, our findings may be more appropriately interpreted as an association analysis within a selected cohort. Fourth, we did not systematically collect detailed information on ICU interventions that may influence lipid metabolism, including corticosteroids, propofol, parenteral nutrition, and hemodilution. Therefore, we cannot completely exclude the possibility that these factors contributed to the observed lipid levels. Detailed information on corticosteroid therapy, including the type, dose, and duration was not systematically collected. Furthermore, immunocompromised status and baseline steroid use may confound the interpretation of HDL-C and clinical outcomes in this study. Finally, given the observational design, our study was intended to evaluate associations between conventional plasma lipid parameters at ICU admission and clinical outcomes, and causality cannot be inferred. Although low HDL-C levels were independently associated with in-hospital mortality, HDL-C may primarily function as a surrogate marker of systemic inflammation and critical illness severity rather than a direct causal factor. The cutoff value of HDL-C was derived and evaluated in the same cohort and has not been independently validated. It should therefore be considered exploratory rather than a clinically established prognostic threshold. HDL-C was analyzed as a continuous variable, and dichotomization may result in loss of information. Further studies using independent cohorts are needed to validate the cutoff and determine whether it provides clinically meaningful prognostic information beyond established severity scores. The biological function of HDL cannot be fully elucidated by measuring absolute plasma HDL-C concentrations alone. Both acute and chronic systemic inflammation, as well as oxidative stress, may induce structural and conformational modifications of HDL particles, leading to potentially impaired antioxidant and anti-inflammatory functions—commonly referred to as dysfunctional HDL—which may further exacerbate inflammation, oxidative stress, and endothelial injury [23,24]. We did not assess genetic factors or the complex heterogeneity of HDL particle composition and function, nor did we investigate the precise cellular and molecular mechanisms linking lipid metabolism to the pathophysiology of sepsis or ARDS. Future studies incorporating functional assays and mechanistic approaches are warranted to better clarify these relationships. Specifically, investigating whether the functional heterogeneity of HDL particles or specific HDL subclasses is causally linked to the pathophysiology of sepsis or ARDS will help determine their potential as early prognostic indicators or therapeutic targets.

## 5. Conclusions

Our findings demonstrate that lower HDL-C levels were associated with hospital mortality in critically ill patients with sepsis. Given that our cohort predominantly consisted of patients with pneumonia and ARDS, these findings should be interpreted within the context of this selected critically ill population and require validation in broader and more heterogeneous sepsis populations.

## Figures and Tables

**Table 1:** Baseline characteristics and clinical variables: patients without ARDS versus with ARDS.

Variables	Without ARDS	With ARDS	*p* Value
	(*n* = 62)	(*n* = 223)	
Age (years)	62.7 ± 14.3	64.7 ± 13.3	0.313
Male (gender)	39 (62.9%)	166 (74.4%)	0.074
Body mass index (kg/m^2^)	22.6 ± 4.5	23.9 ± 4.4	0.044
Infection source			
Bacterial pneumonia	28 (45.2%)	150 (67.3%)	0.001
COVID-19 pneumonia	17 (27.4%)	40 (17.9%)	0.099
Aspiration pneumonia	2 (3.2%)	13 (5.8%)	0.536
Influenza pneumonia	0	10 (4.5%)	0.125
Extrapulmonary sepsis	15 (24.2%)	10 (4.5%)	<0.001
Hypertension	27 (43.5%)	104 (46.6%)	0.666
Diabetes mellitus	29 (46.8%)	77 (34.5%)	0.078
Chronic heart disease	8 (12.9%)	30 (13.5%)	0.910
Chronic lung disease			
COPD	12 (19.4%)	25 (11.2%)	0.091
Asthma	5 (8.1%)	7 (3.1%)	0.088
Interstitial lung disease	2 (3.2%)	10 (4.5%)	0.662
Pulmonary tuberculosis	4 (6.5%)	5 (2.2%)	0.107
Chronic liver disease	8 (12.9%)	23 (10.3%)	0.562
Chronic kidney disease	7 (11.3%)	38 (17%)	0.272
Immunocompromised status ^a^	31 (50%)	105 (47.1%)	0.684
APACHE II score	14.1 ± 5.6	16.7 ± 7.1	0.003
SOFA score	4.6 ± 3.9	8.9 ± 3.8	<0.001
WBC (10^3^/µL)	11.8 ± 5.7	12.1 ± 10.2	0.799
Neutrophil (%)	78.3 ± 18.2	82.8 ± 17.6	0.083
Lymphocyte (%)	8.9 (5.2–14.4)	5.7 (2.9–9.5)	0.012
Creatinine (mg/dL)	0.7 (0.5–1.2)	1.1 (0.5–2.1)	0.006
Bilirubin (total) (mg/dL)	0.7 (0.4–1.1)	0.5 (0.3–0.8)	0.627
Lactate (mg/dL)	11 (7–14.7)	13 (9.5–19.5)	0.849
CRP (mg/L)	78.8 (22.5–133.1)	133.4 (59.2–200.4)	0.001
IL-6 (pg/mL) ^b^	18.1 (4.7–48.4)	39.6 (12.7–171)	0.001
PaO_2_/FiO_2_ (mm Hg)	347.6 ± 88.5	177.2 ± 86.6	<0.001
HDL-C (mg/dL)	31.4 ± 17.7	21 ± 12.6	<0.001
LDL-C (mg/dL)	71.4 ± 40.5	58.2 ± 38.1	0.018
Total cholesterol (mg/dL)	133.4 ± 46.8	114.6 ± 40.6	0.002
Triglyceride (mg/dL)	128.1 ± 80.2	163.3 ± 93.8	0.008
Statin medication	0	17 (7.6%)	
Steroid use	49 (79%)	170 (76.2%)	0.644
Shock status	26 (41.9%)	176 (78.9%)	<0.001
Vasopressor use	21 (33.9%)	167 (74.9%)	<0.001
Mechanical ventilator use	47 (75.8%)	223 (100%)	<0.001
Hospital mortality	12 (19.4%)	96 (43%)	0.001

Data are presented as mean ± standard deviation, count (%) or median (interquartile range). APACHE: Acute Physiology and Chronic Health Evaluation; ARDS: acute respiratory distress syndrome; COPD: chronic obstructive pulmonary disease; COVID-19: coronavirus disease 2019; CRP: C-reactive protein; FiO_2_: fraction of inspired oxygen; HDL-C: high-density lipoprotein cholesterol; IL: interleukin; LDL-C: low-density lipoprotein cholesterol; PaO_2_: partial pressure of oxygen in arterial blood; SOFA: Sequential Organ Failure Assessment; WBC: white blood cells. ^a^ Immunocompromised status included hematological malignancies, solid tumors, sold organ transplantation, long term steroid or immunosuppressant use, and human immunodeficiency virus infection. ^b^ Available for 46 patients without ARDS and 159 patients with ARDS.

**Table 2:** Baseline characteristics and clinical variables: Survivors versus non-survivors.

Variables	Survivors	Non-Survivors	*p* Value
	(*n* = 177)	(*n* = 108)	
Age (years)	63.9 ± 14.7	64.9 ± 11.3	0.543
Male (gender)	125 (70.6%)	80 (74.1%)	0.529
Body mass index (kg/m^2^)	24.3 ± 4.7	22.4 ± 3.7	<0.001
Infection source			
Bacterial pneumonia	105 (59.3%)	73 (67.6%)	0.162
COVID-19 pneumonia	40 (22.6%)	17 (15.7%)	0.160
Aspiration pneumonia	7 (4%)	8 (7.4%)	0.205
Influenza pneumonia	8 (4.5%)	2 (1.9%)	0.328
Extrapulmonary sepsis	17 (9.6%)	8 (7.4%)	0.525
Hypertension	86 (48.6%)	45 (41.7%)	0.255
Diabetes mellitus	69 (39%)	37 (34.3%)	0.423
Chronic heart disease	24 (13.6%)	14 (13%)	0.886
Chronic lung disease			
COPD	27 (15.3%)	10 (9.3%)	0.144
Asthma	10 (5.6%)	2 (1.9%)	0.142
Interstitial lung disease	6 (3.4%)	6 (5.6%)	0.377
Pulmonary tuberculosis	8 (4.5%)	1 (0.9%)	0.160
Chronic liver disease	21 (11.9%)	10 (9.3%)	0.493
Chronic kidney disease	26 (14.7%)	19 (17.6%)	0.514
Immunocompromised status ^a^	69 (39%)	67 (62%)	<0.001
APACHE II score	14.5 ± 6.4	18.9 ± 6.6	<0.001
SOFA score	6.5 ± 3.9	10.3 ± 3.5	<0.001
WBC (10^3^/µL)	11.3 ± 5.8	12.4 ± 10.4	0.329
Neutrophil (%)	81 ± 15.8	83.1 ± 20.7	0.353
Lymphocyte (%)	7.3 (4–11.6)	4.4 (1.9–8.7)	0.044
Serum creatinine (mg/dL)	0.9 (0.5–1.4)	1.1 (0.5–2.7)	0.027
Bilirubin (total) (mg/dL)	0.5 (0.3–0.8)	0.6 (0.3–1)	0.735
Albumin (g/dL)	2.8 ± 0.5	2.6 ± 0.4	0.002
Lactate (mg/dL)	12 (8.9–16.2)	14.4 (10–21.4)	0.029
CRP (mg/L)	123.7 (44.8–191.9)	124.3 (57.1–197.7)	0.617
IL-6 (pg/mL) ^b^	28 (8.2–99.5)	54.2 (17–176)	0.878
PaO_2_/FiO_2_ (mm Hg)	219.2 ± 112.7	185.8 ± 96.8	0.010
HDL-C (mg/dL)	25.2 ± 15.2	20.2 ± 12.7	0.005
LDL-C (mg/dL)	63.8 ± 36.6	56.7 ± 42.3	0.139
Total cholesterol (mg/dL)	123.7 ± 45.1	110.5 ± 37.2	0.012
Triglyceride (mg/dL)	153.6 ± 94.4	159.2 ± 88.3	0.623
Statin medication	12 (6.8%)	5 (4.6%)	0.457
Steroid use	134 (75.7%)	85 (78.7%)	0.561
Shock status	100 (56.5%)	102 (94.4%)	<0.001
Vasopressor use	89 (50.3%)	99 (91.7%)	<0.001
Mechanical ventilator use	162 (91.5%)	108 (100%)	0.001
ARDS	127 (71.8%)	96 (88.9%)	0.001

Data are presented as mean ± standard deviation, count (%) or median (interquartile range). APACHE: Acute Physiology and Chronic Health Evaluation; ARDS: acute respiratory distress syndrome; COPD: chronic obstructive pulmonary disease; COVID-19: coronavirus disease 2019; CRP: C-reactive protein; FiO_2_: fraction of inspired oxygen; HDL-C: high-density lipoprotein cholesterol; IL: interleukin; LDL-C: low-density lipoprotein cholesterol; PaO_2_: partial pressure of oxygen in arterial blood; SOFA: Sequential Organ Failure Assessment; WBC: white blood cells. ^a^ Immunocompromised status included hematological malignancies, solid tumors, sold organ transplantation, long term steroid or immunosuppressant use, and human immunodeficiency virus infection. ^b^ Available for 128 survivors and 77 non-survivors.

**Table 3:** Clinical variables of critically ill sepsis patients stratified by HDL-C at ICU admission.

Variables	HDL-C at ICU Admission		
	High (*n* = 179) (>17.5 mg/dL)	Low (*n* = 106) (≤17.5 mg/dL)	*p* Value
Age (years)	66.1 ± 14	61.2 ± 12.2	0.003
Male (gender)	122 (68.2%)	83 (78.3%)	0.065
Body mass index (kg/m^2^)	23.6 ± 4.7	23.6 ± 4.1	0.957
APACHE II score at day 1	14.9 ± 6.5	18.3 ± 7	<0.001
SOFA score at day 1	6.6 ± 3.8	10.1 ± 3.9	<0.001
WBC (10^3^/µL)	11.4 ± 6	12.2 ± 10.2	0.460
Neutrophil (%)	81.9 ± 16.9	81.7 ± 19.4	0.953
Lymphocyte (%)	7 (3.3–11.5)	4.9 (2.8–9.5)	0.178
Serum creatinine (mg/dL)	0.8 (0.5–1.4)	1.1 (0.5–2.4)	0.118
Bilirubin (total) (mg/dL)	0.5 (0.3–0.7)	0.7 (0.4–1.4)	0.001
Albumin (g/dL)	2.9 ± 0.4	2.5 ± 0.4	<0.001
Lactate (mg/dl)	11.2 (8.7–14.8)	15.9 (11–24.4)	<0.001
CRP (mg/l)	91.6 (30.4–177.6)	158.4 (105.4–219.7)	<0.001
IL-6 (pg/mL) ^a^	21 (7.6–77.8)	66.9 (22.8–229.3)	0.039
PaO_2_/FiO_2_ (mm Hg)	220.5 ± 113.1	182.5 ± 94.4	0.003
HDL-C (mg/dL)	30.6 ± 13.2	10.9 ± 5	<0.001
LDL-C (mg/dL)	70.6 ± 42.1	45 ± 26.2	<0.001
Total cholesterol (mg/dL)	129.1 ± 42.6	101.1 ± 36.7	<0.001
Triglyceride (mg/dL)	134.3 ± 76.1	192 ± 104.9	<0.001
Statin medication	10 (5.6%)	7 (6.6%)	0.726

Data are presented as mean ± standard deviation, count or median (interquartile range). APACHE: Acute Physiology and Chronic Health Evaluation; CRP: C-reactive protein; FiO_2_: fraction of inspired oxygen; HDL-C: high-density lipoprotein cholesterol; ICU: intensive care unit, IL interleukin; LDL-C: low-density lipoprotein cholesterol; PaO_2_: partial pressure of oxygen in arterial blood; SOFA: Sequential Organ Failure Assessment; WBC: white blood cells. ^a^ Available for 133 patients in the high HDL-C group and 72 patients in the low HDL-C group.

**Table 4:** Clinical outcomes as a function of HDL-C at ICU admission in critically ill patients with sepsis.

Outcomes	HDL-C at ICU Admission		
	High (*n* = 179) (>17.5 mg/dL)	Low (*n* =106) (≤17.5 mg/dL)	*p* Value
Mortality			
28-day hospital mortality	31 (17.3%)	38 (35.8%)	<0.001
60-day hospital mortality	50 (27.9%)	49 (46.2%)	0.002
90-day hospital mortality	54 (30.2%)	53 (50%)	<0.001
All cause hospital mortality	55 (30.7%)	53 (50%)	0.002
Shock status	111 (62%)	91 (85.8%)	<0.001
Vasopressor use	99 (55.3%)	89 (84%)	<0.001
Acute kidney injury	51 (28.5%)	46 (43.4%)	0.010
Renal replacement therapy ^a^	25 (14%)	18 (17%)	0.492
Duration of mechanical ventilator (days)	13 (7–27)	15 (9–27)	0.617
Length of ICU stay (days)	15 (9–31)	16 (10–29)	0.743
Length of hospital stay (days)	32 (17–53)	32 (19–48)	0.386

Data are presented as mean ± standard deviation, count or median (interquartile range). HDL-C: high-density lipoprotein cholesterol; ICU: intensive care unit. 28-, 60-, and 90-day mortality were calculated from the date of ICU admission (day 0). ^a^ Patients receiving maintenance hemodialysis before ICU admission were excluded from the renal replacement therapy outcome analysis only, but remained in the overall study cohort.

**Table 5:** Multivariable logistic regression analysis of factors associated with hospital mortality in critically ill patients with sepsis.

Variables	Univariable Analysis		Multivariable Analysis Model	
	OR (95% CI)	*p* Value	Adjusted OR (95% CI)	*p* Value
Age (with each year increase)	1.005 (0.987–1.023)	0.566		
Male (gender)	0.841 (0.491–1.441)	0.529		
Body mass index	0.902 (0.851–0.957)	<0.001	0.913 (0.851–0.978)	0.010
Bacterial pneumonia	1.430 (0.865–2.363)	0.163		
COVID-19	0.640 (0.342–1.197)	0.162		
Hypertension	0.756 (0.466–1.225)	0.256		
Diabetes mellitus	0.816 (0.495–1.344)	0.424		
Immunocompromised status	2.558 (1.564–4.184)	<0.001	2.810 (1.561–5.061)	0.001
SOFA score	1.300 (1.205–1.402)	<0.001	1.290 (1.185–1.405)	<0.001
Serum creatinine	1.148 (1.021–1.290)	0.021		
Albumin	0.364 (0.182–0.730)	0.004		
CRP	1.001 (0.998–1.003)	0.616		
IL-6	1.000 (1.000–1.000)	0.877		
PaO_2_/FiO_2_	0.997 (0.995–0.999)	0.014		
HDL-C	0.973 (0.955–0.992)	0.006	0.955 (0.921–0.990)	0.013
LDL-C	0.995 (0.988–1.002)	0.143		
Total cholesterol	0.992 (0.986–0.998)	0.013		
Triglyceride	1.001 (0.998–1.003)	0.622		
Steroid use	1.186 (0.668–2.107)	0.561		

CI: confidence interval; COVID-19: coronavirus disease 2019; CRP: C-reactive protein; FiO_2_: fraction of inspired oxygen; HDL-C: high-density lipoprotein cholesterol; IL: interleukin; LDL-C: low-density lipoprotein cholesterol; OR: odds ratio; PaO_2_: partial pressure of oxygen in arterial blood; SOFA: Sequential Organ Failure Assessment. For the continuous variables, the odds ratio indicates that the odds of hospital mortality increases or decreases per unit increase of these variables.

**Table 6:** Sensitivity analyses of the association between HDL-C and hospital mortality in critically ill patients with sepsis.

Model	Additional Covariates	Adjusted OR for HDL-C (95% CI)	*p* Value
Primary model (*n* = 285)	Baseline covariates	0.955 (0.921–0.990)	0.013
Sensitivity model 1 (*n* = 285)	+ CRP	0.959 (0.934–0.995)	0.021
Sensitivity model 2 (*n* = 205)	+ IL-6	0.954 (0.925–0.983)	0.017
Sensitivity model 3 (*n* = 285)	+ PaO_2_/FiO_2_	0.960 (0.936–0.995)	0.019

CI confidence interval, CRP C-reactive protein, FiO_2_ fraction of inspired oxygen, HDL-C high-density lipoprotein cholesterol, IL interleukin, OR odds ratio, PaO_2_ partial pressure of oxygen in arterial blood, SOFA Sequential Organ Failure Assessment. The primary model included age, gender, body mass index, immunocompromised status, SOFA score, serum creatinine, albumin, and HDL-C based on clinically driven selection. Sensitivity analyses were performed by additionally adjusting for CRP, IL-6, or PaO_2_/FiO_2_, as specified for each model. Missing data were handled using a complete-case approach, and the sample size for each model is reported.

## Data Availability

The datasets used or analyzed in the study are available from the corresponding author upon reasonable request.

## Abbreviations

The following abbreviations are used in this manuscript:

APACHE	Acute Physiology and Chronic Health Evaluation
AKI	acute kidney injury
ARDS	acute respiratory distress syndrome
BMI	body mass index
CI	confidence interval
COVID-19	coronavirus disease 2019
CRP	C-reactive protein
HDL	high-density lipoprotein
HDL-C	high-density lipoprotein cholesterol
ICU	intensive care unit
IL	interleukin
LDL-C	low-density lipoprotein cholesterol
OR	odds ratio
ROC	receiver operating characteristic
SOFA	sequential organ failure assessment.
